# gH625-liposomes as tool for pituitary adenylate cyclase-activating polypeptide brain delivery

**DOI:** 10.1038/s41598-019-45137-8

**Published:** 2019-06-24

**Authors:** Giuseppina Iachetta, Annarita Falanga, Yves Molino, Maxime Masse, Francoise Jabès, Yasmine Mechioukhi, Vincenza Laforgia, Michel Khrestchatisky, Stefania Galdiero, Salvatore Valiante

**Affiliations:** 1 Department of Biology, University of Naples “Federico II”, Via Cinthia, 80126 Naples, Italy; 2 Department of Agricultural Sciences, University of Naples “Federico II”, Via Università, 100, 80055 Portici, Italy; 3 CiRPEB- University of Naples “Federico II”, Via Mezzocannone 16, 80134 Napoli, Italy; 4 Vect-Horus SAS, Marseille, France; 5 Aix-Marseille Univ, CNRS, INP, Inst Neurophysiopathol, Marseille, France; 6 Department of Pharmacy - University of Naples “Federico II”, Via Mezzocannone 16, 80134 Napoli, Italy; 7 National Institute of Biostructures and Biosystems (INBB), V. le Medaglie d’Oro, 00136 Rome, Italy

**Keywords:** Microscopy, Blood-brain barrier, Molecular medicine, Neurological disorders

## Abstract

The blood-brain barrier (BBB) regulates the traffic of molecules into the central nervous system (CNS) and also limits the drug delivery. Due to their flexible properties, liposomes are an attractive tool to deliver drugs across the BBB. We previously characterized gH625, a peptide derived from *Herpes simplex* virus 1. The present study investigates the efficiency of liposomes functionalized on their surface with gH625 to promote the brain uptake of neuroprotective peptide PACAP (pituitary adenylate cyclase-activating polypeptide). Using a rat *in vitro* BBB model, we showed that the liposomes preparations were non-toxic for the endothelial cells, as assessed by analysis of tight junction protein ZO1 organization and barrier integrity. Next, we found that gH625 improves the transfer of liposomes across endothelial cell monolayers, resulting in both low cellular uptake and increased transport of PACAP. Finally, *in vivo* results demonstrated that gH625 ameliorates the efficiency of liposomes to deliver PACAP to the mouse brain after intravenous administration. gH625-liposomes improve both PACAP reaching and crossing the BBB, as showed by the higher number of brain cells labelled with PACAP. gH625-liposomes represent a promising strategy to deliver therapeutic agents to CNS and to provide an effective imaging and diagnostic tool for the brain.

## Introduction

The high blood-brain barrier (BBB) impermeability and selectivity prevent the transport of many therapeutic molecules into the brain and thus makes ineffective their administration for the treatment of neurological disorders^1,2^. In the last years several strategies for brain drug delivery have been developed^3–5^. Nanocarrier-mediated strategy is emerging as a non-invasive and effective method to explore for treatment of neurological diseases^6,7^. Among nanocarriers, liposomes are considered promising tools for drug delivery to the brain^8–11^. Liposomes are able to incorporate and deliver hydrophilic, lipophilic, and hydrophobic molecules, having lipid and water compartments. They show excellent biocompatibility and biodegradability, low toxicity, and controlled drug release^7,12–19^. Furthermore, their surface can be functionalized with different ligands to target specific sites and facilitate site-specific delivery^20–22^. Functionalization with biologically active ligands may improve their binding and transport across the BBB^23,24^.

Recently, cell-penetrating peptides (CPPs), short cationic and/or amphipathic peptides, have been used to facilitate drug delivery^25–28^. CPPs can transport many types of macromolecules through the membrane bilayer *in vitro* and *in vivo*^29–31^. Membranotropic CPPs undergo direct translocation across membranes making the transported cargo immediately available in the cytosol, avoiding endosomal entrapment and lysosome degradation^32^. The gH625 peptide was identified as a membrane-perturbing domain in glycoprotein H (gH) of *Herpes simplex* virus 1, that can traverse the membrane bilayer and deliver several molecules across cell membranes *in vitro*^33–37^. The surface functionalization of polystyrene nanoparticles with gH625 enhances their transport throught an *in vitro* model of the BBB^38^. Furthermore, gH625 can be efficiently internalized by neuroblastoma and astrocytoma cell lines and it crosses the BBB and reaches the brain when injected in rats, despite liver filtration^39^. We recently showed that gH625 promotes the fusion of giant unilamellar vesicles and multivalency is key for internalization^40^. Based on this background, we evaluated the efficacy of liposomes functionalized with gH625 to transport a therapeutic molecule *in vitro* across a rat BBB model and *in vivo* in mice. In our experiments, liposomes were loaded with pituitary adenylate cyclase-activating polypeptide (PACAP), a neurotrophic and neuroprotective peptide proposed for treatment of central nervous system (CNS) injuries, stroke, and neurodegenerative diseases^41,42^. PACAP is a member of the vasoactive intestinal peptide/secretin/growth hormone-releasing hormone/glucagon superfamily^43,44^. PACAP showed neuroprotective effects in neurodegeneration models of cerebral ischemia and brain injuries^41,44–49^ and in other neurological diseases such as Parkinson’s disease^50–54^ and Alzheimer’s disease^46,50,54–59^. Nevertheless, use of PACAP in clinical practice presents some limitations because of its low stability in human plasma^60^, rapid degradation^61^ and peripheral actions^62–64^. Therefore, new strategies to improve PACAP stability and its delivery to the CNS are necessary. Here we developed liposomes loaded with PACAP27, that we fuctionalized on their surface with gH625 and we characterized by dynamic light scattering (DLS). We performed studies on a rat *in vitro* BBB model^65,66^ to assess liposome cellular toxicity, uptake by and transport across endothelial cell monolayers. Finally, after mice tail intravenous administration, we evaluated the efficacy of the functionalized liposomes *in vivo*, by analysing PACAP brain distribution with light sheet fluorescence microscopy.

## Results

### Characterization of liposomes

**Figure 1:**
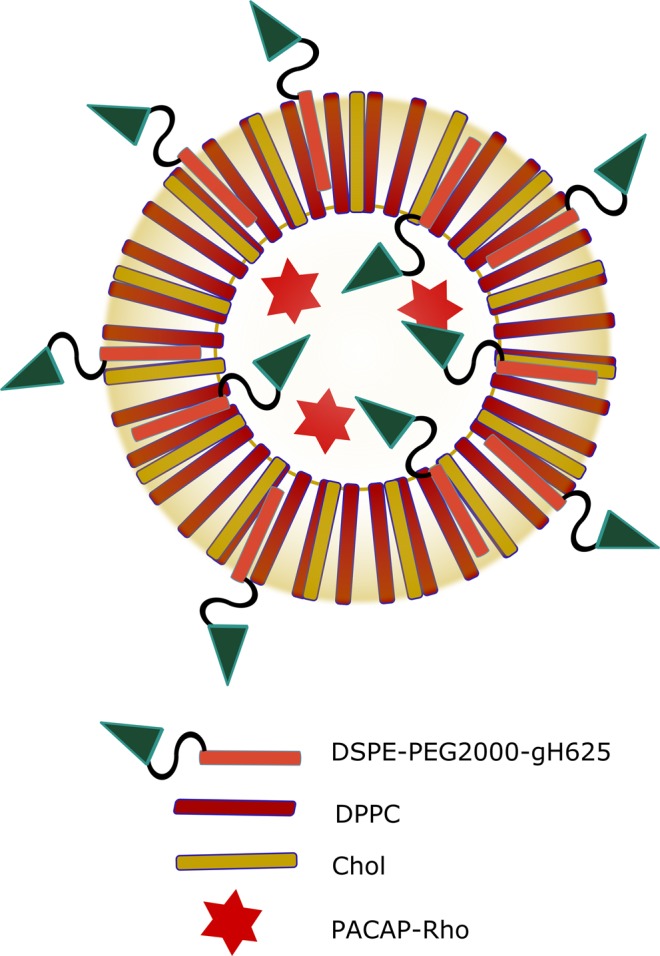
Scheme of liposomes functionalizated on the surface with gH625 and loaded with PACAP27.

Liposomes loaded with PACAP-Rho and functionalized on their surface with gH625 (Fig. 1) were characterized. The sequences of synthesized peptides are reported in Table 1. The hydrodynamic diameters (DH) and polydispersity index (PDI) of all liposomes were measured using dynamic light scattering (DLS). Three independent experiments were performed for each sample and each measurement was performed at least in triplicate. All liposome solutions present a monomodal distribution with a polydispersity index (PDI) <0.2 indicating a narrow and homogenous size distribution **(**Table 2**)**.

**Table 1:** Sequence and molecular weight of peptides used.

Peptide	Sequence	Molecular weight
gH625Cys	Ac-HGLASTLTRWAHYNALIRAF-Cys-CONH_2_	2442.77
DSPE-PEG2000-gH625	Ac-HGLASTLTRWAHYNALIRAF-Cys-PEG2000DSPE	5383.77
PACAP27	HSDGIFTDSYSRYRKQMAVKKYLAAVL-CONH_2_	3147.63
Rho-PACAP27	RHO-HSDGIFTDSYSRYRKQMAVKKYLAAVL-CONH_2_	3561.16

**Table 2:** Liposomes size and zeta potential analysis.

Liposomes	Averange Size (nm)	PDI
Liposomes loaded with PACAP	127.10 ± (10.65)	0.16 ± (0.14)
Liposomes loaded with PACAP and gH625 on the surface	173.60 ± (5.33)	0.23 ± (0.01)

### Uptake of liposomes by brain microvascular endothelial cells (BMECs)

**Figure 2:**
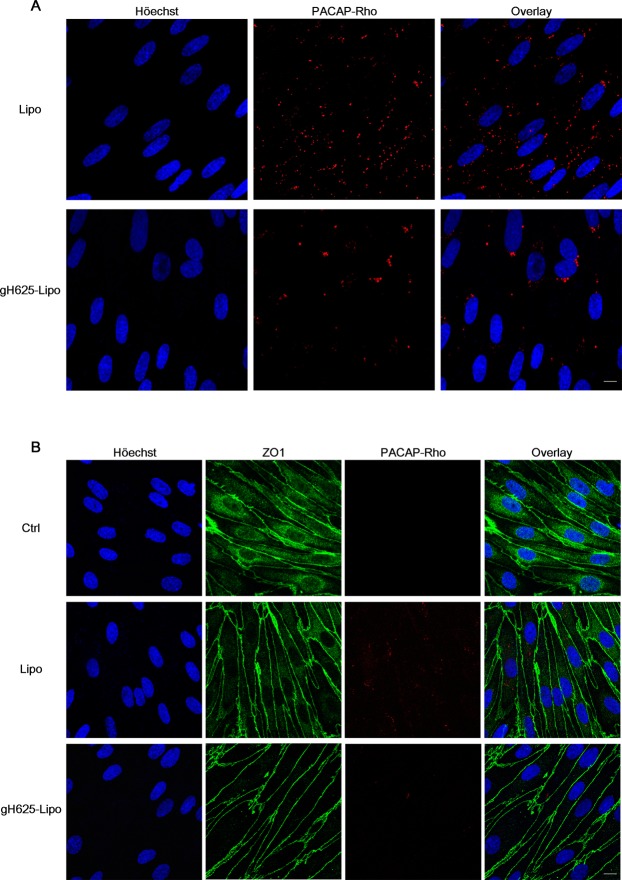
Liposome uptake and organization of the tight junction protein ZO1 in BMEC monolayers after liposomes incubation. (**A**) Photomicrographs of BMEC monolayers subjected to incubation of Lipo and gH625-Lipo loaded at 5 µM PACAP-Rho for 60 min on live cells (Scale bars, 10 µm). Nuclei were stained with Höechst. (**B**) Photomicrographs of BMEC monolayers subjected to incubation of Lipo and gH625-Lipo loaded at 5 µM PACAP-Rho for 60 min on live cells. Cells were fixed, permeabilized and stained with an anti-ZO1 antibody (Scale bars, 10 µm). Nuclei were stained with Höechst. Note that the photomicrographs show no significant difference between the Lipo and gH625-Lipo groups compared to control.

Following 60 min incubation on live brain microvascular endothelial cells (BMECs) of Lipo and gH625-Lipo loaded at 5 µM PACAP-Rho, the photomicrographs showed a very low presence of PACAP-Rho in both experimental classes (Fig. 2A). Note that at 1 µM, PACAP-Rho was not detectable within BMECs (not shown). The representative fluorescence images at 5 µM of PACAP-Rho showed that the non-functionalized liposomes undergo more BMEC uptake compared to the gH625-functionalized liposomes (Fig. 2A).

To observe the effect of liposomes on the cellular organization of BMEC monolayers, we assessed immunofluorescence of the tight junction protein ZO1. Confocal microscopy analysis showed that 60 min incubation on live BMEC monolayers at the highest concentration of 5 µM PACAP-Rho, Lipo and gH625-Lipo caused no qualitative change in ZO1 distribution and presumably no toxicity (Fig. 2B). Note that for both experimental classes, PACAP-Rho fluorescence in BMECs is lower than that in Fig. 2A, presumably due to the membrane permeabilization step with Triton X100 surfactant for ZO1 immunostaining that might disintegrate part of liposomes.

### Barrier integrity and transport of liposomes across BMEC monolayers

**Figure 3:**
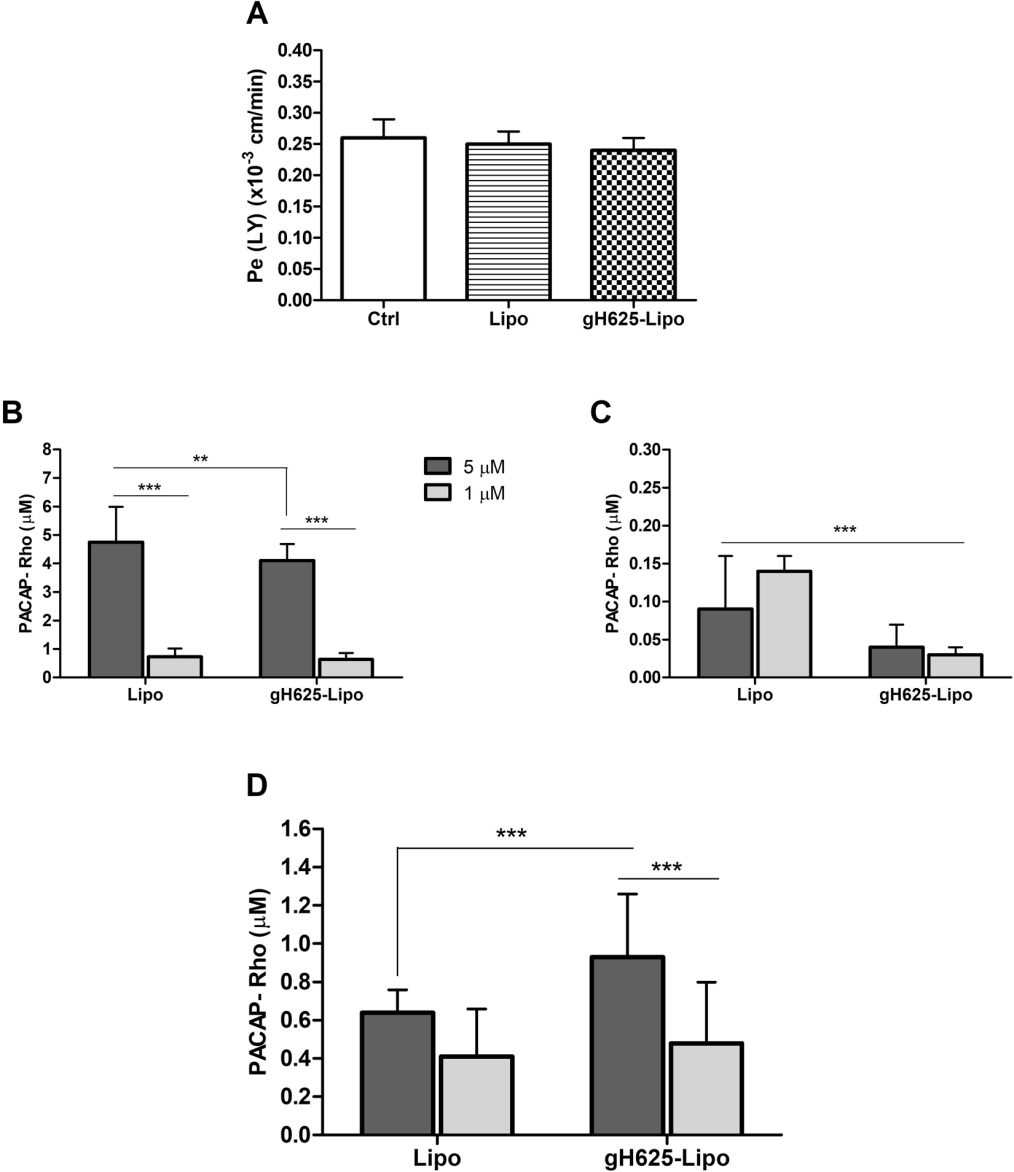
Barrier integrity and liposome uptake by and transport across BMEC monolayers. (**A**) To verify the barrier integrity of the BBB *in vitro* model during liposome incubation, Lipo and gH625-Lipo loaded at 5 µM PACAP (without rhodamine) were co-incubated with LY in the luminal compartment in contact with BMEC monolayers for 60 min at 37 °C. After this time, the medium of the abluminal compartment was collected and LY fluorescence was quantified by fluorimetric analysis with a spectrofluorimeter (λ_ex_ 430/485 nm; λ_em_ 535 nm). The results are expressed in permeability for LY or Pe_(LY)_ in 10^−3^ cm/min. Values represent means ± SD of 3 independent experiments. Note that the Pe_(LY)_ results show no significant impact of both liposome preparations on BMEC monolayers integrity compared to control (Ctrl: cells alone). (**B**) Amounts of PACAP-Rho in the luminal compartment after incubation with Lipo and gH625-Lipo loaded at 1 µM and 5 µM PACAP-Rho for 60 min at 37 °C; (**C**) PACAP-Rho in the BMEC lysate; (**D**) PACAP-Rho in the abluminal compartment. Values represent means ± SD of 5 independent experiments (**P < 0.005; ***P < 0.0005).

Before we performed the transport experiments, the cellular toxicity of liposomes was further assessed by monitoring the barrier integrity of the BBB *in vitro* model. Lipo and gH625-Lipo loaded at 5 µM PACAP were co-incubated with LY for 60 min at 37 °C on BMECs monolayers. Results showed that the LY permeability coefficient values (Pe) were on average 0.24 ± 0.03 × 10^−3^ cm/min for both liposome preparations, and similar to the control without liposomes that is below the threshold of LY paracellular leakage. These results showed that neither Lipo alone nor gH625-Lipo loaded at 5 µM PACAP altered the barrier integrity of the BMEC monolayers (Fig. 3A).

**Figure 4:**
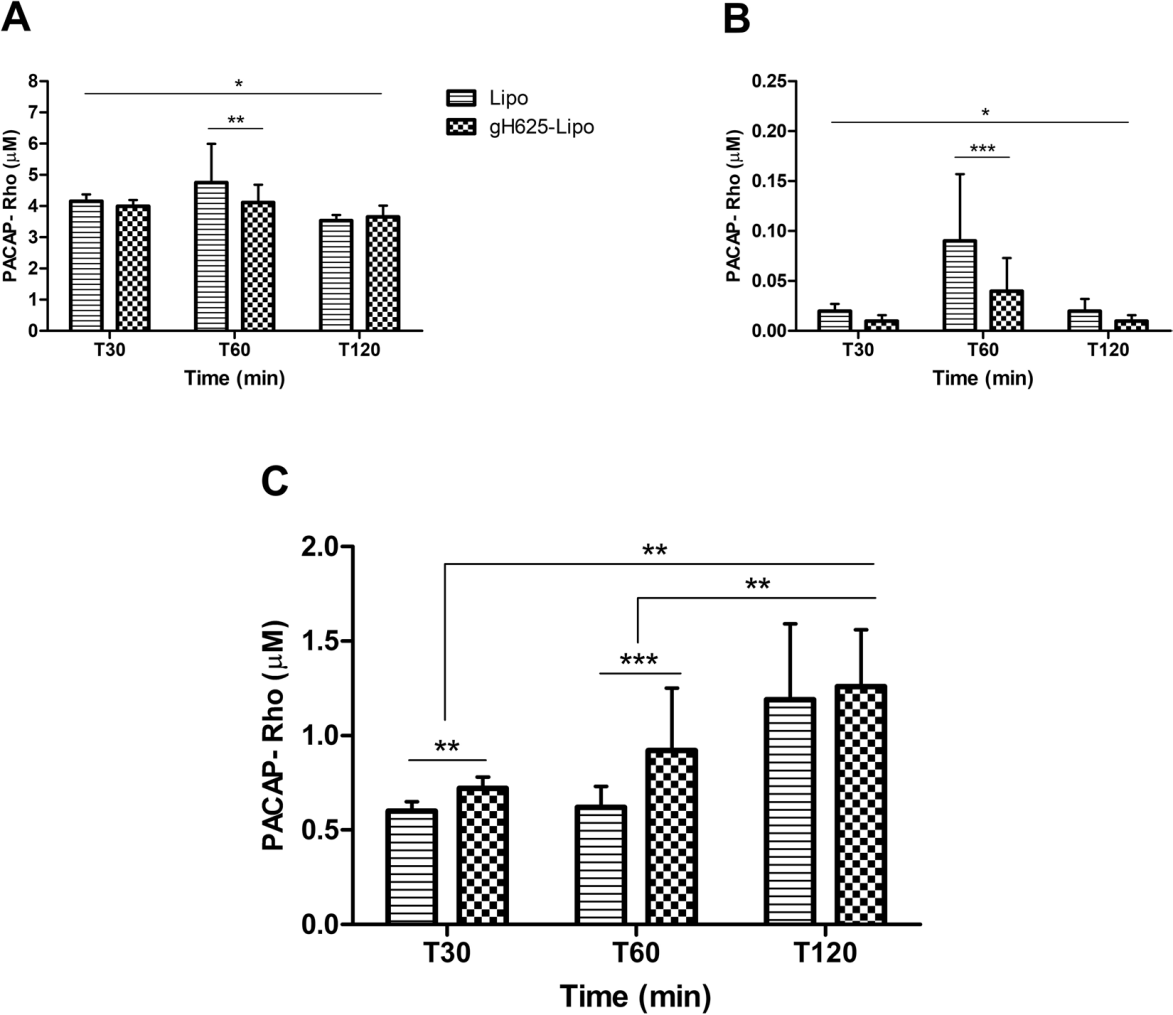
Evaluation of uptake by and transport across BMEC monolayers of liposomes at different times. Quantification of Lipo and gH625-Lipo loaded at 5 µM PACAP-Rho in different compartments of the *in vitro* BBB model after incubation in the luminal compartment in contact with BMEC monolayers for 30, 60 and 120 min at 37 °C. (**A**) Amounts of PACAP-Rho in the luminal compartment; (**B**) PACAP-Rho in the BMEC lysate; (**C**) PACAP-Rho in the abluminal compartment. Values represent means ± SD of 5 independent experiments (*P < 0.05; **P < 0.005; ***P < 0.0005).

**Figure 5:**
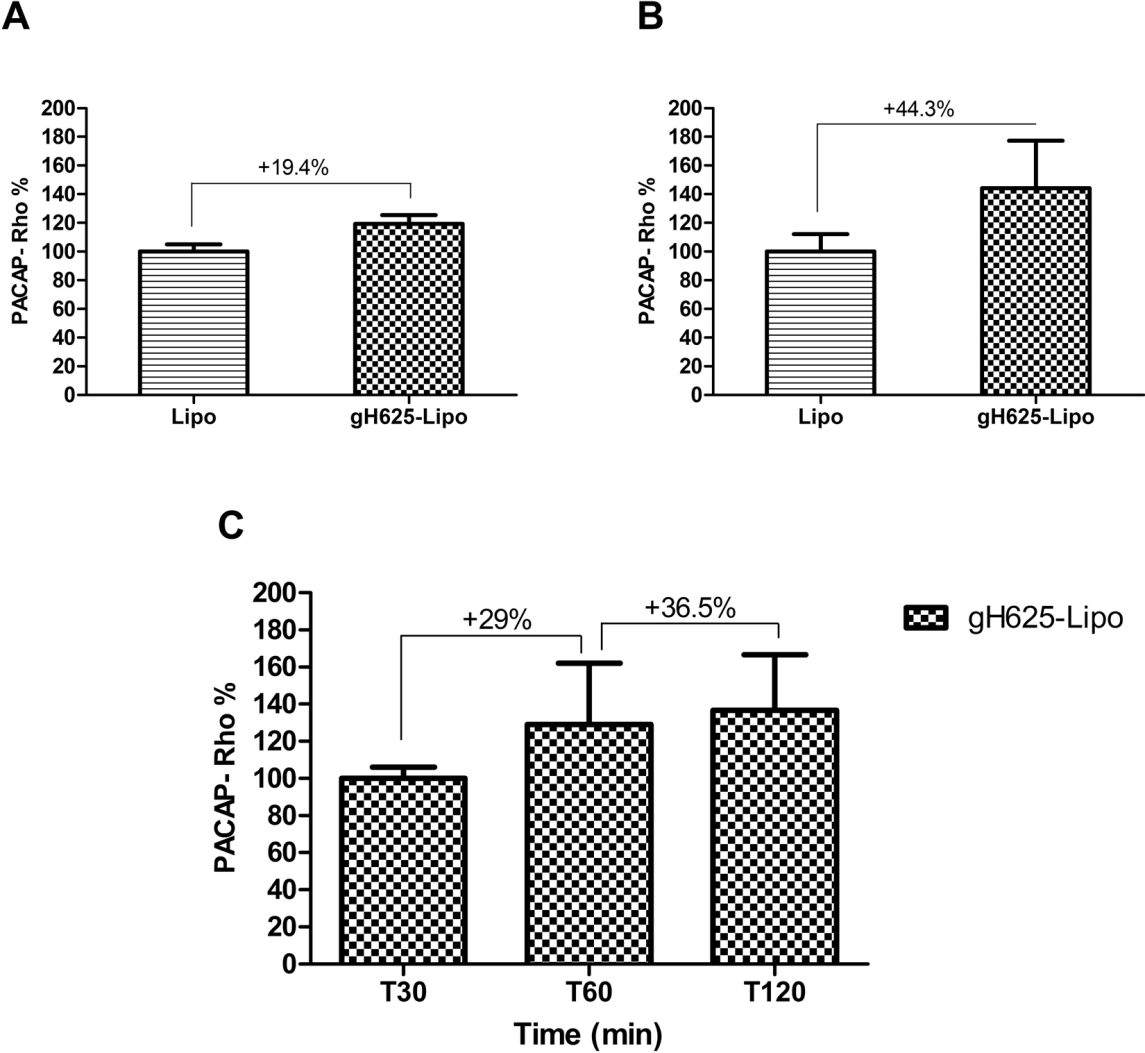
Data analysis of liposome transport experiments: transport of gH625-Lipo expressed as variation with respect to Lipo (100%). (**A**) after 30 min; (**B**) after 60 min; (**C**) transport of gH625-Lipo expressed as variation with respect to 30 min of incubation (100%). Values are generated from the transport experiments showed in Fig. 4.

For transport studies, gH625-Lipo and Lipo were incubated at 5 µM and 1 µM PACAP-Rho on BMEC monolayers. After incubation, the amount of dye was determined in the cell lysate, and in the luminal and abluminal compartments and compared to the initial amount of dye incubated. Standard curves were performed for each class of liposomes and for free PACAP-Rho and used to obtain accurate concentrations of PACAP-Rho in all compartments of the *in vitro* BBB model. Results showed that the amount of PACAP-Rho in the luminal compartment after 60 min decreased compared to the initial concentration of PACAP-Rho (Fig. 3B). The amount of PACAP-Rho in BMECs was very low for both liposomes but significantly higher for Lipo than for gH625-Lipo (Fig. 3C). At lower concentration (1 µM) the amount of PACAP-Rho in the abluminal compartment was very low (around 0.41 µM), without significantly difference between the two classes of liposomes. Liposome transport across BMEC monolayers increased with concentration but was statistically significant only for the gH625-Lipo class. At higher concentration (5 µM), the amount of PACAP-Rho that crossed the monolayer was higher for gH625-Lipo than unfunctionalized liposomes (0.93 and 0.64 µM, respectively) (Fig. 3D). The experiments of transport at different times, at 5 µM concentration, show that after each time of incubation the amount of PACAP-Rho in the luminal compartment decreased compared to the initial concentration of PACAP-Rho (Fig. 4A). PACAP-Rho in BMECs was very low for both liposomes at each time point, with significant differences only at 60 min (Fig. 4B). The data from the abluminal compartment indicated that liposome transport was time-dependent. The amount of PACAP-Rho that crossed BMEC monolayers was higher for gH625-Lipo than Lipo also in the first 30 min of incubation (0.72 and 0.60, respectively) (Fig. 4C). After 120 min, the amount of PACAP-Rho detected in the abluminal compartment was higher compared to the 30 min time point for both liposomes but without significant difference between gH625-Lipo and Lipo (Fig. 4C). Data analysis on the abluminal compartment at 5 µM showed that the amount of PACAP-Rho which crosses the BMEC monolayers after 30 min of incubation is 19.4% higher when transported by gH625-Lipo than Lipo (Fig. 5A) and 44.3% higher after 60 min (Fig. 5B). Furthermore, PACAP-Rho transported across BMEC monolayers by gH625-Lipo increased by 29% from 30 min to 60 min and by 36.5% from 60 min to 120 min (Fig. 5C).

### Brain distribution of PACAP-Rho in mice after *i*.*v*. administration of liposomes

**Figure 6:**
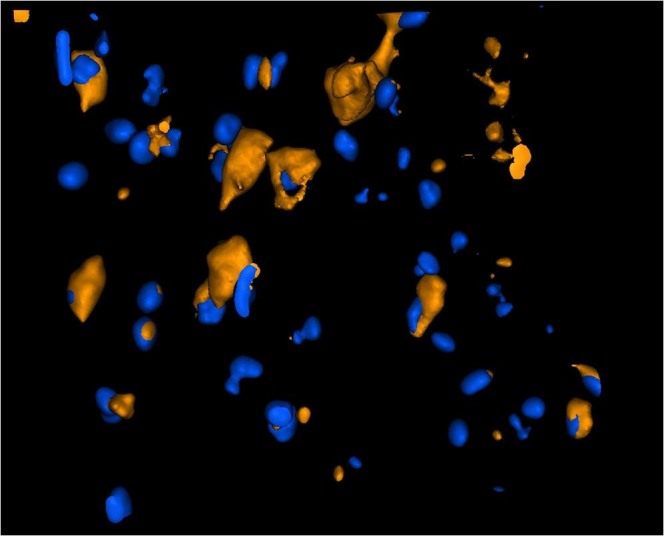
Brain 3D rendering of gH625-Lipo after *i*.*v*. administration in mice. Representative 3D rendering of gH625-Lipo brain parenchyma z-stack acquisition: PACAP-Rho labelled cytoplasms (orange) and cell nuclei (blue) are showed.

**Figure 7:**
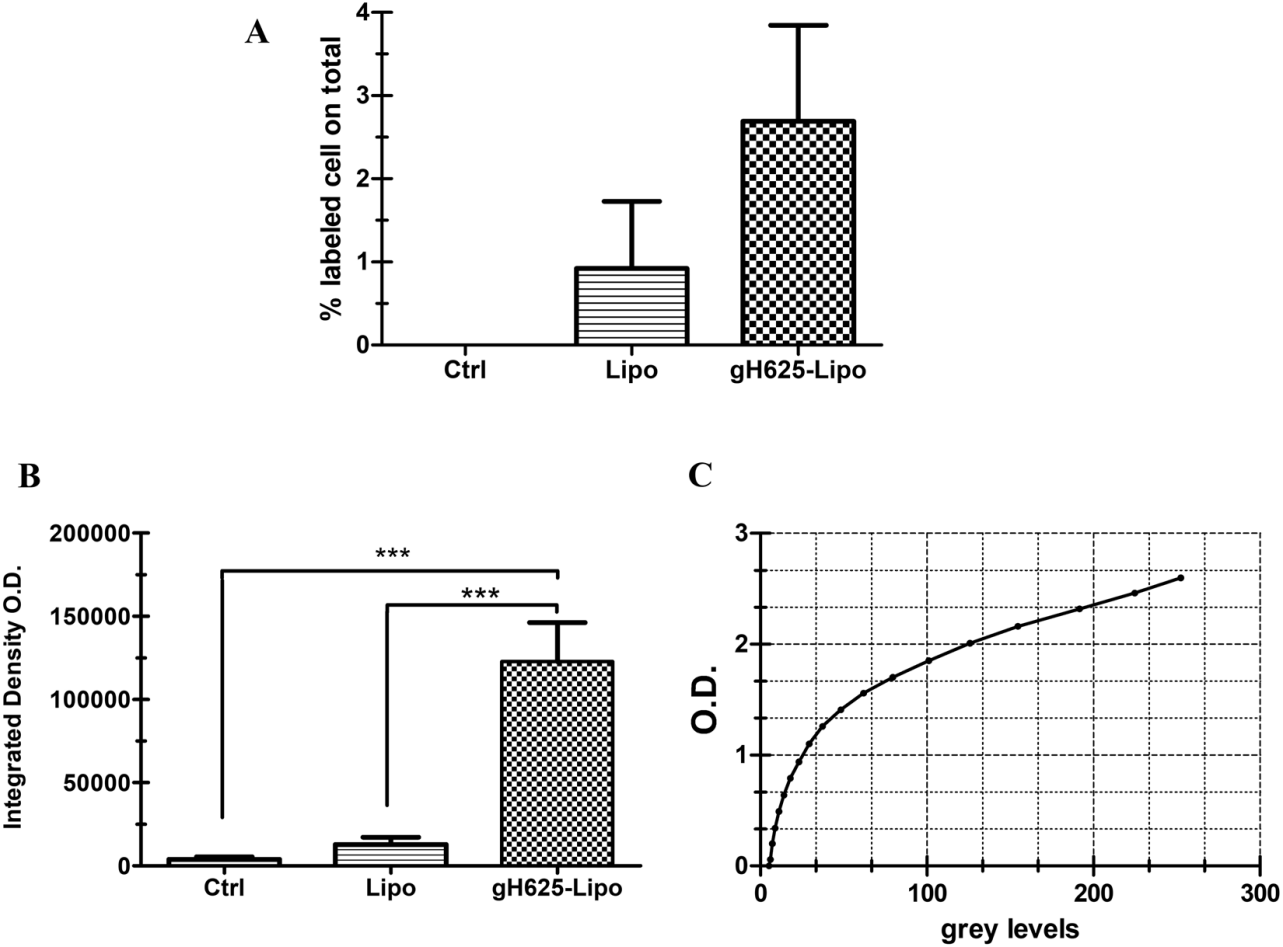
Brain distribution of PACAP-Rho in mice after *i*.*v*. administration of liposomes. (**A**) Percentage of PACAP-Rho labelled cells in brain volumes analysed after treatment with Lipo and gH625-Lipo; (**B**) Integrated density of brain volumes after treatment with Lipo and gH625-Lipo; (**C**) calibration curve. ***P < 0.001; **P < 0.01; *P < 0.05.

For light sheet fluorescence microscopy analysis, the total volume (µm^3^) of brains and the total number of cells analysed was reported in Table 3. The results showed an increase of labelled cells within the region of interest (ROI) (Fig. 6). In particular, the administration of Lipo led to release of PACAP-Rho in 1.34% of cells (Fig. 7A); the percentage of cells labelled with PACAP-Rho in the brain of gH625-Lipo treated animals was doubled (Fig. 7A). Fluorescence measured in control brains was considered as background. Integrated density was calculated to detect the amount of fluorescence within the ROI (Fig. 7B). 3D brain volume reconstruction of control, Lipo and gH625-Lipo experimental groups are showed in Supplementary Material section (Movies 1–3).

**Table 3:** Total volume of brains and number of cells analysed.

	Control	Lipo	gH625-Lipo
**Volume (µm** ^**3**^ **)**	6.26*10^8^	7.88*10^8^	8.30*10^8^
**Cell number**	10581	14618	15629

**Figure 8:**
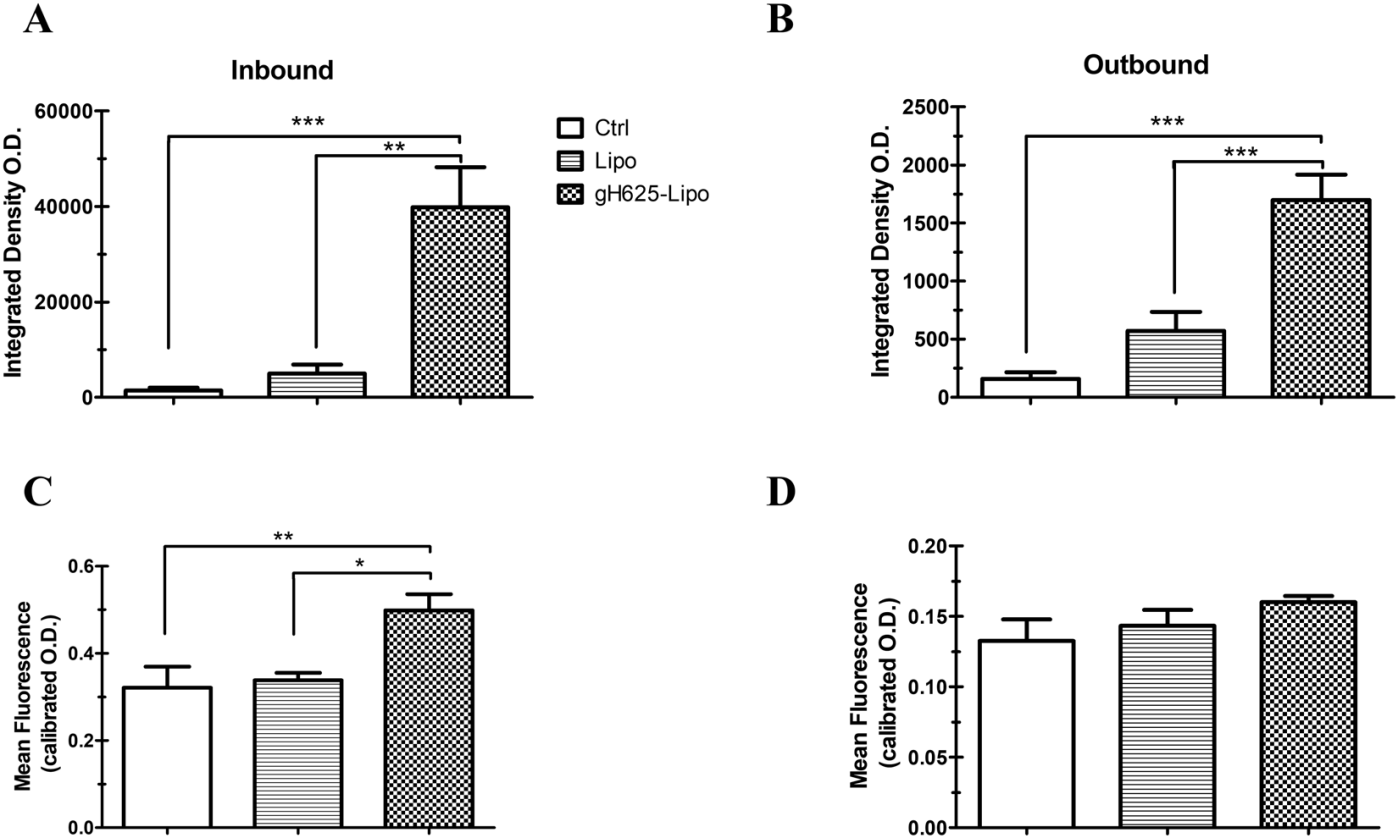
Analysis of PACAP-Rho distribution along BBB capillaries and brain parenchyma after *i*.*v*. administration in mice. Analysis of fluorescence parameters inside (inbound column, **A**,**C**) and outside (outbound column **B**,**D**) BBB capillaries in mouse brain for gH625-Lipo, Lipo and NaCl injected controls. Measure of integrated density O.D. and mean fluorescence O.D. within the blood capillaries of the entire brain volume region of interest (**A**,**C**). Measure of integrated density O.D. and mean fluorescence O.D. outside the blood capillaries of the entire brain volume region of interest (**B**,**D**). Values represent means ± SEM, (*P < 0.05; **P < 0.005; ***P < 0.0005).

The analysis of PACAP-Rho distribution in mice brain BBB vessels is showed in Fig. 8. The inbound fluorescence, which means fluorescence signal within the blood vessels, is significantly higher in gH625-Lipo samples than in control and Lipo class both as mean and integrated density values (Fig. 8A,C). The outbound fluorescence, which means fluorescence outside blood vessels, is significantly higher in gH625-Lipo samples than in control and Lipo class as integrated density values show (Fig. 8B) but only slightly higher as mean (Fig. 8D).

## Discussion

The BBB is a highly selective vascular system; this is beneficial for neural cells that are protected from toxic molecules and pathogens but renders brain drug administration difficult or ineffective. Overcoming this limitation is mandatory and different strategies are evaluated to ameliorate brain drug delivery. Here we present results obtained with liposomes functionalized with a membranotropic CPP (gH625) to deliver the neuroprotective PACAP peptide across the BBB, both *in vitro* and *in vivo*.

Our *in vitro* studies demonstrated that gH625 functionalized liposomes are better nanocarriers for BBB drug delivery than liposomes. The uptake studies first showed the ability of gH625 to favour low retention of PACAP in the BMECs compartment compared to unfunctionalized liposomes, likely changing the mechanism of liposomes uptake in the BMECs. It is of note that gH625 liposomes appeared devoid of toxic effects as indicated by ZO1 immunostaining that shows no alteration of cellular organization of BMEC monolayers; this latter result is also supported by the barrier integrity assay, suggesting that gH625 does not impact on the liposome safety profile. gH625 ameliorates the transport of liposomes across BMEC monolayers and increases significantly the amount of PACAP that crosses these monolayers. This occurs in a time dependant manner, suggesting that no receptor is involved in the transport process. Further, we demonstrated that gH625 allows liposomes to transport higher amounts of PACAP beyond the BMEC monolayers at every time point considered.

The *in vivo* experiments in mice showed that the gH625-liposomal formulation loaded with PACAP improved both PACAP reaching and crossing the BBB, with a higher number of PACAP positive brain cells compared to non-functionalized liposomes. This result demonstrates that gH625 improves brain delivery of liposomes.

The light sheet fluorescence microscopy analysis showed that gH625 liposomes allowed PACAP-Rho to reach the BBB more efficiently than non-functionalized liposomes; gH625 liposome showed a 2-fold increase in the number of labelled parenchyma cells. Although we did not perform any cito-typing of these cells, the morphology of the labeled cells strongly resembles that of neurons. To our knowledge, this is the first report of effective brain cell labeling beyond the BBB, following *in vivo* administration of a functionalized nanovector.

We also showed that gH625 liposomes ameliorate the PACAP-Rho reaching of the BBB (about 4-fold) compared to non-functionalized liposomes. Furthermore, gH625 liposomes increase PACAP-Rho capability to overstep the BBB (about 3-fold), compared to non-functionalized liposomes.

Hence, our analysis of the PACAP-Rho distribution in the BBB brain vessels and brain parenchyma, demonstrates that gH625 consistently helps PACAP-Rho to reach and cross the BBB and to enter neuron-like cells.

Although the exact molecular mechanism for the entry of gH625 remains to be established, the results of this study show for the first time that a membranotropic peptide derived from *Herpes simplex* virus type 1 can improve the transport of liposomes across the BBB endothelium, both *in vitro* and *in vivo* in mice.

## Materials and Methods

### Materials

Fmoc-protected amino acid derivatives, coupling reagents, and Rink amide *p*-methylbenzhydrylamine (MBHA) resin were purchased from Calbiochem-Novabiochem (Laufelfingen, Switzerland). All phospholipids were purchased from Avanti Polar Lipids (Alabaster, AL). Reagents (piperidine and pyridine) for solid-phase peptide synthesis were purchased from Fluka (Milan, Italy). Trifluoroacetic acid and acetic anhydride were from Applied Biosystems (Foster City, CA). H_2_O, N,N dimethylformamide, and CH_3_CN were supplied by Labscan Ltd. (Dublin, Ireland). Dulbecco’s Modified Eagle’s Medium/Nutrient Mixture F-12, fetal bovine serum, penicillin-streptomycin, HEPES buffer, gentamicin and bFGF were purchased from Invitrogen (Carlsbad, CA, USA). Cell culture flasks (75 cm^2^), 12-well cell culture plates, collagen type IV mouse and fibronectin human plasma were obtained from Becton-Dickinson (Franklin Lakes, NJ, USA). Bovine serum from platelet poor plasma (Clinisciences, Nanterre, France). Lucifer Yellow CH, dilithium salt, hydrocortisone and puromycin were from Sigma-Aldrich (St Louis, MO, USA). Polyethylene hanging cell inserts for 12-well plates (porosity: 1 µm; surface: 1.1 cm^2^) were purchased from Millipore (Burlington, MA, USA).

### Animals

Procedures involving animals conform to National and European regulations (EU directive N°2010/63) and to authorizations granted to our animal facility (N°C13 055 08), to the INP laboratory and to the project (N°00757.02) by the Ethics Committee of the Aix Marseille University and the French Ministry of Research. All efforts were made to minimize animal suffering and reduce the number of animals used. We used Swiss CD1 mice 35 g and Wistar rat 5–6 weeks (Elevage Janvier, St Berthevin, France).

### Peptide synthesis

The gH625Cys and PACAP27 peptides were synthesized using a standard solid-phase Fmoc (9-fluorenylmethoxycarbonyl) method as previously reported^67^ and were obtained with good yields (30–40%). PACAP27 was prepared with and without Rhodamine labeled (5(6)-Carboxytetramethylrhodamine N-succinimidyl ester). Labeling was performed on resin-bound peptides as previously reported^68^. Peptides were fully deprotected and cleaved from the resin with trifluoroacetic acid. The crude peptides were precipitated with icecold ethyl ether, filtered, dissolved in water, lyophilized, and purified by preparative reverse-phase HPLC. The samples were eluted with a solvent mixture of H_2_O and 0.1% trifluoroacetic acid (solvent A) and CH_3_CN and 0.1% trifluoroacetic acid (solvent B). A linear gradient of 20–80% solvent B over 20 min at a flow rate of 20 ml/min was employed. The collected fractions were lyophilized to dryness and analyzed by mass spectrometry. For the synthesis of DSPE-PEG2000-gH625, DSPE-PEG2000-Mal (1 eq) was reacted with gH625-Cys (1 eq) in DMF containing triethylamine (5 eq) for 24 h. After the completion of the reaction, the solvent was evaporated, and the residue was redissolved in water, lyophilized and analyzed by RP-HPLC. Products were analyzed by MALDI-TOF mass spectrometry (MALDI-TOF MS). The peptides were stored at −20 °C until used.

### Preparation and characterization of liposomes

Large unilamellar vesicles (LUV) consisting of DPPC/Chol (70/30 mol/mol) were prepared as previously reported (Galdiero *et al*., 2005)^67^. Lipid concentrations of liposome suspensions were determined by phosphate analysis^69^ Briefly, various amounts of lipids and when necessary DSPE-PEG2000-gH625 and/or PACAP-Rho were dissolved in chloroform. The solvent was then removed with a nitrogen gas stream and the sample was lyophilized overnight. The lipid film was suspended in buffer by vortexing to produce LUVs, freeze-thawed eight times and then extruded 10 times through polycarbonate membranes with 0.1 µm diameter pores (Northern Lipids). PACAP-Rho encapsulated in liposomes was quantified by ultracentrifugation followed by HPLC analysis. The hydrodynamic diameters (DH) and polydispersity index (PDI) of PACAP-Rho loaded liposomes (Lipo) and PACAP-Rho loaded gH625-liposomes (gH625-Lipo) were measured using dynamic light scattering (DLS) (Malvern Zetasizer Nano ZS, Malven, UK). The analysis was performed with He–Ne laser 4 mW operating at 633 nm at scattering angle fixed at 173 °C and at 25 °C.

### Rat *in vitro* BBB model

Primary rat brain microvascular endothelial cells (BMEC) and primary astrocytes necessary for setting up a rat *in vitro* BBB co-culture model were obtained as reported (Molino *et al*.^65,66^. Briefly, primary cultures of BMECs, from 5- to 6-week-old Wistar rats, were seeded in the luminal compartment of twelve-well plate polyethylene insert filters (Merck Millipore, Billerica, MA, USA), pre-coated with collagen type IV and fibronectin (BD Biosciences, Franklin Lakes, NJ, USA) to establish the endothelial cell monolayers. Astrocyte primary cultures were prepared from the cerebral cortex of newborn Wistar rats and seeded in the bottom of the twelve-well plates to establish the co-culture with the endothelial cell monolayers in endothelial cell media (ECM) containing DMEM/F12 supplemented with 20% bovine platelet poor plasma derived serum (Alfa Aesar, Ward Hill, MA, USA), basic fibroblast growth factor (bFGF) 2 ng/mL, heparin 100 μg/ mL, gentamycin 50 μg/mL, HEPES 2.5 mM, and hydrocortisone 500 nM (Life Technologies, Carlsbad, CA, USA). Under these conditions, the BMEC monolayers differentiate, express junction-related proteins within 3 days, and remain optimally differentiated during three more days.

### Immunofluorescence and confocal microscopy analysis

#### Uptake of liposomes by BMECs

Lipo and gH625-Lipo loaded at 1 µM and 5 µM PACAP-Rho, were incubated for 60 min at 37 °C on BMEC monolayers. After incubation, the cells were fixed for 15 min with a 4% (w/v) paraformaldehyde solution followed by cutting of the insert membranes and nuclei staining with Höechst 33258 (Invitrogen, Carlsbad, CA, USA). The membranes were washed three times with phosphate-buffered saline and then mounted on microscope slides. The analyses of slides were performed using confocal microscopy LSM 780 (Zeiss, Jena, Germany) equipped with a 63x oil-immersion objective, and ZEN Software 2012. Images were acquired using filters for rhodamine channel (λ_ex_544 nm; λ_em_584 nm) and Höechst channel (λ_ex_350 nm; λ_em_461 nm).

#### ZO1 Immunofluorescence

Zonula occludens (ZO1)-1 immunofluorescence was also performed to evaluate tight junction morphology and liposome toxicity. Lipo and gH625-Lipo, loaded at 5 µM PACAP-Rho, were incubated on BMECs for 60 min at 37 °C. After incubation, the insert filters with BMECs were washed twice with phosphate-buffered saline and fixed with a 4% (w/v) paraformaldehyde solution for 15 min. Cells were then permeabilized for 10 min with PBS 0.1% Triton X100 followed by washing with PBS twice. Blocking was performed using 3% BSA for 30 min followed by PBS wash and incubation with the ZO1 antibody (Rabbit anti-ZO1 Zymed 61–7300) diluted 1:200 in a solution of 1% BSA for 60 min at room temperature. After two washes, cells were incubated with AlexaFluor 488 (1:800) secondary antibodies and Höechst (1:1000) for 30 min at room temperature, followed by three washes with PBS. Finally, cells were mounted with ProLong® Gold Antifade Mountant (Life Technologies) and visualized under confocal microscopy (LSM 780; Carl Zeiss, Jena, Germany) equipped with a 63x oil-immersion objective by using filters for Alexa 488 channel (λ_ex_544 nm; λ_em_584 nm) and Höechst channel (λ_ex_ 350 nm; λ_em_ 461 nm).

### Barrier integrity assay

In order to control the BMEC monolayer integrity of the *in vitro* BBB model and absence of toxicity of liposomes, transport assays were performed as previously described (Molino *et al*.,^65,66^). Briefly, the BMEC monolayers were gently washed with pre-warmed DMEM/F12 without phenol red and transferred to clean twelve-well plates. Lucifer yellow (LY; Sigma-Aldrich) was co-incubated with Lipo or gH625-Lipo, at 5 µM PACAP (without rhodamine) in the luminal compartment of the culture system, in contact with BMECs, for 60 min at 37 °C. After this time, the medium of the abluminal compartment was collected and fluorescence was quantified by fluorimetric analysis with the SpectraMax® M5e (Molecular Devices, Sunnyvale, CA, USA) equipped with a fluorescence filter (λ_ex_ 430/485 nm; λ_em_ 535 nm). The results represent the LY paracellular leakage from the luminal to the abluminal compartment and are expressed in permeability coefficient (Pe) in 10^−3^ cm/min. Barrier integrity was validated for Pe_(LY)_ below 0.6 × 10^−3^ cm/min.

### Quantification of liposome uptake by and transport across BMEC monolayers

To evaluate their transport across BMEC cell monolayers, Lipo and gH625-Lipo were incubated for 60 min at 37 °C at 5 µM and 1 µM PACAP-Rho in the luminal compartment of twelve-well plate polyethylene insert filters. After incubation, the medium was collected from the luminal and abluminal compartments from each group and cell monolayers rinsed twice with phosphate-buffered saline and then lysed with 500 μL/insert PBS 0.1% Triton X100 in order to release the internalized peptide. The fluorescence of medium and cell lysates was quantified by SpectraMax® M5e (Molecular Devices, Sunnyvale, CA, USA) equipped with a fluorescence filter (λ_ex_ 544 nm; λ_em_ 584 nm). Furthermore, liposomes loaded with 5 µM PACAP-Rho were incubated for 30, 60 and 120 min at 37 °C to evaluate the passage across BMEC monolayers at different times. Three BBB *in vitro* models were used for each time condition. Fluorescence values were normalized using the equation of the standard curve for each class of liposomes. The standard curve was obtained using liposomes with different concentrations of PACAP-Rho loaded, i.e., 0.156 μM, 0.312 μM, 0.625 μM, 1.25 μM, 2.5 μM, and 5 μM. As negative controls, the DMEM/Ham’s F12 without phenol red was used. Each experiment was repeated five times.

### Distribution of PACAP-Rho in the cortex of mice after intravenous (i.v.) administration of liposomes

Mice of mean 35 g were maintained in a temperature-controlled room at 24 °C, with 12-hours light/dark cycle and with free access to a standard rodent diet and water before experiments. Animals were randomly divided in 3 groups, each consisting of 5 mice. Unfuctionalized and gH625 functionalized liposomes loaded with 100 µM PACAP-Rho were intravenously injected in the tail vein (bolus of 150 μl). All liposome formulations contained the same lipid concentration and 0.9% saline solution were used as negative control. After 90 min from liposomal injection, all mice were intraperitoneally anesthesied at a letal dose of Ketamine (100 mg/kg) and Xylasine (10 mg/kg) solution. Mice were then intracardially perfused with 0.9% saline solution following by a 4% paraformaldehyde solution. Brains were then postfixed overnight at 4 °C with 4% paraformaldehyde. Then after 3 washes in PBS 1X, brains were prepared for light sheet fluorescence microscopy analysis.

#### Brain clearing and light sheet fluorescence microscopy analysis

For light sheet fluorescence analysis treated mouse brains were first dissected sagittally, and each part was dissected coronally approximately to 1.0 mm from Bregma (http://mouse.brain-map.org/). Samples were stained with Höechst for 4 hours and then cleared by ScaleA2 protocol, an urea-based solution that renders mouse brains transparent^70^. ScaleA2 solution was prepared using 4 M urea, 10% (wt/vol) glycerol and 0.1% (wt/vol) Triton X-100. After two weeks, samples were mounted in the Z1 Light Sheet Fluorescence Microscope (Zeiss) in the imaging chamber. Samples were overviewed and centered by led light illumination for acquisition of coronal stacks, and illuminated by λ_ex_ 488 nm, λ_ex_ 405 nm and λ_ex_ 561 nm laser sources, respectively; corresponding fluorescence signals were detected through a 20X NA = 1.0 Zeiss water immersion objective and images captured by PCO.Edge sCMOS water cooled cameras. Raw data were acquired and analyzed by ZEN 2012 software (Zeiss); Eight-bit fluorescence image stacks were calibrated using Rodbard NIH Image optical density (O.D.) curve between 0 and 2,58 and then analysed with Fiji software;^71^ nuclei were counted in each volume stack using the ROI manager3D (v.3.92) and mean (the fluorescence of samples as average gray value within the sample) and integrated density (as the product of mean grey value of ROI and ROI area) evaluated on thresholded volume stacks to avoid background artifacts. To estimate the amount of PACAP-Rho that crossed the BBB, we considered the mean and the integrated density of specific ROIs: in detail, to evaluate the PACAP-Rho which reached the BBB without crossing it, the selected ROI was considered as the inside of the brain blood vessels (inbound), exclusively; then, on the non-zero thresholded volume stacks, an inversion of selected ROI was carried out to obtain the ROI where to calculate the amounts of PACAP-Rho that crossed the BBB (outbound), exclusively.

### Statistical analysis

Data *in vitro* are expressed as mean ± SD and the statistical comparisons were carried out between the groups using two sample *t*-test assuming unequal variances. Data *in vivo* are expressed as mean ± SEM and one-way Anova with Bonferroni’s multiple comparison test was used for the experiments in mice. A significance value of at least *P* < 0.05 was accepted and considered as relevant.

## Supplementary information

Supplementary info

Movie 1

Movie 2

Movie 3
