# Supplementary material for: gH625-liposomes as tool for pituitary adenylate cyclase-activating polypeptide brain delivery
Source: Sci Rep. 2019 Jun 24;9:9183. doi: 10.1038/s41598-019-45137-8 (PMC6591382; doi:10.1038/s41598-019-45137-8)
Supplement: Supplementary file 1 — Supplementary info [file 41598_2019_45137_MOESM1_ESM.docx]

**gH625-liposomes as tool for pituitary adenylate cyclase-activating polypeptide brain delivery**

*Giuseppina Iachetta^1^, Annarita Falanga^2,3^, Yves Molino^4^, Maxime Masse^4^, Francoise Jabès^4^, Yasmine Mechioukhi^4^, Vincenza Laforgia^1^, Michel Khrestchatisky^5^, Stefania Galdiero^3,6^, Salvatore Valiante^1,7*^*

^1^Department of Biology, University of Naples “Federico II”– Via Mezzocannone, 8, 80134, Napoli, Italy

^2^Department of Agricultural Sciences, University of Naples “Federico II”-Via Università, 100, 80055, Portici, Italy

^3^CiRPEB- University of Naples “Federico II”, Via Mezzocannone 16, 80134, Napoli, Italy

^4^Vect-Horus SAS, Marseille France

# ^5^Aix-Marseille Univ, CNRS, INP, Inst Neurophysiopathol, Marseille, France

^6^Department of Pharmacy - University of Naples “Federico II”, Via Mezzocannone 16, 80134, Napoli, Italy

^7^National Institute of Biostructures and Biosystems (INBB), V. le Medaglie d’Oro, 00136, Rome, Italy

**Supplementary legend**

**Movie 1:** Representative brain volume 3D reconstruction of control group. Very low background is showed.

**Movie 2:** Representative brain volume 3D reconstruction of Lipo group. Blood capillaries and some cells are labelled for PACAP-Rhod.

**Movie 3:** Representative brain volume 3D reconstruction of gH625-Lipo group. Blood capillaries and numerous cells are labelled for PACAP-Rhod.
